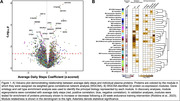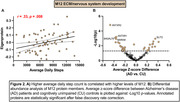# Network‐based Plasma Proteomics Reveals Molecular Overlap Between Physical Activity and Dementia Risk

**DOI:** 10.1002/alz.089188

**Published:** 2025-01-09

**Authors:** Rowan Saloner, Emily W. Paolillo, Claire J. Cadwallader, Anna M. VandeBunte, Coty Chen, Tony Wyss‐Coray, Joel H. Kramer, Kaitlin B. Casaletto

**Affiliations:** ^1^ Memory and Aging Center, UCSF Weill Institute for Neurosciences, University of California, San Francisco, San Francisco, CA USA; ^2^ Wu Tsai Neurosciences Institute, Stanford University, Stanford, CA USA

## Abstract

**Background:**

Physical activity (PA) is associated with lower dementia risk; however, underlying molecular pathways are poorly understood. We leveraged large‐scale plasma proteomics to identify biological signatures of objectively‐monitored PA in cognitively unimpaired (CU) older adults and cross‐validated signatures in independent exercise intervention and Alzheimer’s disease (AD) cohorts.

**Method:**

Discovery cohort included 65 CU adults (mean_age_ = 76.6; 60% female; 34% PET Ab+) from the UCSF Memory and Aging Center with plasma assayed on SomaScan v4.1 (7,288 proteins). 30‐days of observational Fitbit^TM^ monitoring quantified PA (average daily steps). Generalized linear models (GLMs) examined individual protein correlates of PA, adjusting for age and sex. Weighted gene network analysis assembled proteins into unbiased modules of protein co‐expression, which were annotated for gene ontology and cell‐type enrichment. Modules were tested for overrepresentation of PA‐related proteins using Fisher’s exact tests with false discovery rate (FDR) correction. To validate PA pathways, modules were cross‐referenced with published plasma exercise intervention data (Robbins et al., 2023) of proteins that changed following a 20‐week endurance training intervention. To establish clinical relevance, PA‐related proteins from discovery analyses were compared between CU (N=189) and AD patients (N=55) from an independent cohort with plasma SomaScan (Stanford ADRC).

**Result:**

Discovery: PA related to 568 proteins in GLMs. Of 32 protein modules identified from network analyses, six showed overrepresentation of PA‐related proteins. Module 12 (M12) positively associated with PA and harbored the largest proportion of PA‐related proteins. M12 was enriched for extracellular matrix (ECM) and nervous system development pathways with endothelial, neuronal, and oligodendrocyte cell type markers. Validation: M12 was significantly enriched for ECM‐related proteins that increased in the exercise intervention. Four proteins from M12 were significantly decreased in AD plasma compared to CU after FDR correction, including highly‐connected ‘hub’ proteins anthrax toxin receptor cell adhesion molecules 1 and 2 (ANTXR1, ANTXR2) and matrix remodeling associated 8 (MXRA8).

**Conclusion:**

Large‐scale analysis of the plasma proteome highlights ECM and neurodevelopmental pathways that increase with PA and decrease in AD. Cross‐validation with independent cohorts identified a biological link between PA and dementia risk. PA‐related ‘hub’ proteins, such as ANTXR1, ANTXR2, and MXRA8, may represent key molecular targets for dementia prevention.